# Temporal Lobectomy Evidence for the Role of the Amygdala in Early Emotional Face and Body Processing

**DOI:** 10.1523/ENEURO.0114-24.2024

**Published:** 2025-02-14

**Authors:** Eleanor Moses, Jenna Scambler, Jessica Taubert, Ada H. Y. Lo, Kate Thompson, Beatrice de Gelder, Alan J. Pegna

**Affiliations:** ^1^School of Psychology, The University of Queensland, St Lucia, Queensland 4067, Australia; ^2^Department of Psychology, Queensland Health, Brisbane, Queensland 4000, Australia; ^3^Psychology Department, Royal Brisbane Women’s Hospital, Herston, Queensland 4029, Australia; ^4^Department of Cognitive Neuroscience, Faculty of Psychology and Neuroscience, Maastricht University, Maastricht 6211 LK, The Netherlands

**Keywords:** amygdala, bodily emotion, EEG, emotion, facial emotion, temporal lobe resection

## Abstract

The amygdala is believed to make invaluable contributions to visual emotion processing. Yet how this subcortical body contributes to emotion perception across time is contended. Here, we measured differences in the perceptual processing of emotional stimuli after unilateral temporal lobe and amygdala resection (TLR) in humans, using EEG. Through mass univariate analysis of brain activity, we compared responses to fearful and neutral faces (left TLR *N* = 8, right TLR *N* = 8, control *N* = 8), and fearful and neutral bodies (left TLR *N* = 9, right TLR *N* = 9, control *N* = 9). We found that TLR impaired the early-stage perceptual processing of emotional stimuli seen in the control group. Indeed, in controls a heightened responses to fearful faces was found in the 140–170 ms time window, over temporoparietal electrodes. This effect was also present in the left TLR group but disappeared in the right TLR group. For emotional bodies, brain activity was differentially sensitive to fearful stimuli at 90–120 ms in the control group, but this effect was eliminated in both TLR groups. Collectively, these results reveal the amygdala contributes to the early stages of perceptual processing that discriminate emotional stimuli from neutral stimuli. Further, they emphasize the unique role of the right medial temporal structures such as the amygdala in emotional face perception.

## Significance Statement

This research is the first case to date to measure the electrophysiological correlates of emotional expressions represented by bodies after medial temporal resection as compared with healthy controls. It is also the first instance of the use of mass univariate analysis to assess EEG data to emotional content after TLR. This work sheds light on the potential impact of medial temporal lobe resection on early affective visual stimuli processing. Importantly, this research also indicates the integral role that resected regions like the amygdala play in early affective processing of human forms.

## Introduction

The processing of emotional facial expressions plays a crucial role in navigating our social world. Extensive research has focused on investigating the neural mechanisms that encode and drive attention toward emotional content in visual scenes. The amygdala—a neural structure located within the medial temporal lobe—plays a pivotal role in perceptual processing of emotional stimuli. However, its exact contribution from a dynamic perspective is debated because the amygdala may receive visual input through multiple pathways. For instance, while information is conveyed to the amygdala from the primary visual system via the temporal cortex, it may also receive information more rapidly via a subcortical route believed to bypass the lateral geniculate nucleus (LGN), facilitating the rapid detection and prioritization of emotional input including emotional facial expressions (LeDoux, 1994; Tamietto and de Gelder, 2010).

In neuropsychology, cases of blindsight—where damage to the primary visual cortex has caused hemianopia or complete blindness—have been used to demonstrate that amygdala activation does not require cortical input. For example, when fearful faces are presented to the blind field, both performance (Tamietto and de Gelder, 2008) and early electrophysiological responses are spared (Cecere et al., 2014). This residual function has been attributed to the subcortex because neuroimaging studies have shown that the amygdala continues to respond to emotional faces in blindsight patients (Pegna et al., 2005; Burra et al., 2019). Further, behavioral studies have found that the facilitatory effect of emotional faces presented in the blind field is absent in cases of comorbid hemianopia and subcortical path disruption following pulvinar loss (Bertini et al., 2018). While neuropsychological studies have provided clear evidence that the amygdala plays a pivotal role in the processing of emotional facial expressions, the operationalization of this structure and its function in perceptual processing remains contended (Pessoa and Adolphs, 2010; McFadyen et al., 2017).

Emotional signals are not provided by facial expressions alone. For example, both bodily and facial expressions represent salient stimuli that are associated with action tendencies (de Gelder et al., 2004; de Gelder, 2006). Negative bodily expressions have both been shown to attract attention and elicit valence-matched facial responses in participants (Kret et al., 2013). Regarding the presentation of emotional bodies to blind visual hemifields, the available evidence suggests that emotion perception is intact, with expressions of fear evoking matched corrugator supercilia responses and degrees of pupil dilation irrespective of whether the stimuli are fearful faces or fearful bodies (Tamietto et al., 2009). It has also been shown that emotional bodies presented to the blind field evoke selective activation of the middle temporal visual area (MT) and pulvinar nucleus of the thalamus (de Gelder and Hadjikhani, 2006), bilateral superior colliculus, amygdala, and right fusiform gyrus, as well as cortical motion areas (Van Den Stock et al., 2011). Thus, even without input from the visual cortex, information about emotional bodies propagates throughout the brain. But are emotional bodies processed by the same mechanisms as emotional faces? Although bodily and facial expressions drive activity in the amygdala, fusiform gyrus, and the superior temporal sulcus (STS; van de Riet et al., 2009; Li et al., 2023), a key question that is yet to be resolved is whether the role of the amygdala is the same for both faces and bodies.

A population of interest are those with pharmacological resistant temporal lobe epilepsy, who undergo surgical resection of the focal seizure origin. The resection site can include the medial structures such as the amygdala as well as a portion of the anterior temporal lobe (TLR; temporal lobe resection). This population is noted to have impaired processing of emotional stimuli. TLR populations have shown blunted behavioral responses to affective stimuli (Burton et al., 2003). In neuroimaging studies, right TLR resection has been linked to reduced cortical responses to faces (Reisch et al., 2022a) and emotional scenes (Reisch et al., 2022b). While these observations indicate that the amygdala plays a role in emotion processing, it is not yet clear how TLR patients respond to emotional bodily expressions.

Here we use EEG to investigate the speed with which TLR patients process facial and bodily expressions. Two studies to date have investigated EEG responses to affective imagery after TLR (Framorando et al., 2021; Mielke et al., 2022). Both conducted ERP amplitude analysis on components related to emotion processing and attention (such as the P1, N170, EPN, and LPP). Mielke et al. (2022) comparing responses to affective scenes for a right TLR population found no emotion modulation to early components (P1 and N1). In addition, Framorando et al. (2021) compared left and right TLR groups and found the typical N170 emotion modulation was absent in the right TLR group. However, it remains unknown whether the temporal lobe resection equally impacts the neural processing of facial and bodily expressions. This question is the motivation for the current study; here, we compare the processing of facial emotion and bodily emotion in two groups, patients with left TLR (LTLR) and right TLR (RTLR). Our goal is to shed light on the contribution of the amygdala to the processing of emotional bodies and faces from a dynamic perspective and to probe possible functional lateralization, by exploring ERP responses in TLR patients with unilateral amygdala resection. We approach this using mass univariate analysis (MUA), which provides better global spatial and temporal resolution than conventional techniques and allows for the concurrent analysis of all channels.

## Materials and Methods

### Participants

All clinical TLR participants underwent resection of the unilateral amygdala. In some cases, portions of the temporal lobe and the hippocampus were also resected (Tables 1, 2).

**Table 1.** Clinical participants face task

Gender	Age	Task	Surgery laterality	Temporal pole resection	Hippocampal resection	Age of seizure onset	Years since surgery	Seizure free
M	28	1-back	L	No	Yes	7	14	Yes
F	42	1-back	L	No	Yes	12	6	Yes
F	26	1-back	L	Yes	Yes	1	14	Yes
F	28	1-back	L	Yes	Yes	N/A	2	Yes
M	49	1-back	L	Yes	Yes	15	14	Yes
F	47	1-back	L	Yes	Yes	12	8	Yes
M	47	FER	L	Yes	Yes	20	2	Yes
M	34	FER	L	Yes	No	17	4	Yes
M	35	FER	L	Yes	No	25	4	Yes
M	46	1-back	R	No	Yes	25	13	No
F	53	1-back	R	No	No	31	0.25	Yes
F	32	1-back	R	Yes	No	26	1	N/A
F	23	1-back	R	Yes	Yes	5	1	N/A
F	22	1-back	R	Yes	Yes	7	3	Yes
F	56	FER	R	Yes	Yes	2	0.25	No
M	35	FER	R	Yes	Yes	11	10	Yes
F	46	FER	R	Yes	Yes	14	17	Yes
F	31	FER	R	Yes	Yes	27	11	Yes

Clinical information for lobectomy participants for the face task. The focal seizure origin for all patients was temporal. All TLR patients underwent unilateral resection of the amygdala on either the left (L) or right (R). In some instances, resection sites also included portions of the temporal pole and/or the hippocampus. Inclusion of the temporal pole and hippocampus in resection is indicated in the 5th and 6th columns (marked by Yes or No). Patients in the facial emotion sample completed either a one-back task (as published in Framorando et al., 2021) or a facial emotion recognition task (represented as FER). The task completed (1-back or FER) is represented in the 3rd column.

**Table 2.** Clinical participants body task

Gender	Age	Included in face sample	Surgery laterality	Temporal pole resection	Hippocampal resection	Age of seizure onset	Years since surgery	Seizure free
M	46		L	Yes	Yes	23	1	Yes
M	47	Y	L	Yes	Yes	20	2	Yes
F	45		L	Yes	Yes	35	2	Yes
M	34	Y	L	Yes	No	17	4	Yes
F	55		L	Yes	No	2	8	No
M	35	Y	L	Yes	No	25	4	Yes
F	47		L	Yes	Yes	12	1	No
M	25		L	Yes	No	<1	0.5	Yes
M	46	Y	R	No	Yes	25	13	No
F	27		R	Yes	No	11	1	No
F	29		R	Yes	No	5	2	Yes
F	53	Y	R	No	No	31	0.25	Yes
F	47		R	Yes	Yes	30	3	Yes
F	32	Y	R	Yes	No	26	1	NA
F	23	Y	R	Yes	Yes	5	1	NA
F	45		R	Yes	Yes	31	0.5	Yes

Clinical information for lobectomy participants for the body task. The focal seizure origin for all patients was temporal. All TLR patients underwent unilateral resection of the amygdala on either the left (L) or right (R). In some instances, resection sites also included portions of the temporal pole and/or the hippocampus. Inclusion of the temporal pole and hippocampus in resection is indicated in the 5th and 6th columns (marked by Yes or No). All participants completed the same bodily emotion recognition task.

#### Facial emotion task

For the facial emotion task, data from two lobectomy experiments were combined. The first (published in Framorando et al., 2021) was a previously collected dataset and included an LTLR group (4 female; age *M* = 36.67, SD = 9.58) and RTLR group (4 female; age *M* = 38.00, SD = 11.84). The second newly acquired group included 7 lobectomy participants, 3 LTLR (0 female; age *M* = 38.67, SD = 5.91) and 4 RTLR (3 female; age *M* = 38.50, SD = 11.71). The total set comprises 9 LTLR (4 female; age *M* = 37.33, SD = 8.59) and 9 RTLR (7 female; age *M* = 38.22, SD = 11.79).

The healthy control group was acquired to match the size and proportion of the clinical group that had completed each of the two tasks [9 total, 5 completing the task from Framorando et al. (2021); 4 female, age *M* = 23.40, SD = 8.87, and 4 completing the task as described below; 1 female, age *M* = 33.50, SD = 9.91]. The total set of 9 healthy control participants included 5 females, with age *M* = 27.89, SD = 10.61.

#### Bodily emotion task

Twenty-four participants additionally completed a bodily emotion task. There were 8 per group (LTLR, RTLR, and control). The LTLR group was composed of 3 females, with age *M* = 41.75, SD = 8.98. The RTLR group was composed of 7 females, with age *M* = 37.75, SD = 10.49. Of the control group, 1 was female, with age *M* = 39.50, SD = 10.26.

### Stimuli and procedure

These studies were approved by a number of governing ethical bodies. The participant set from Framorando et al. (2021) was approved by the Ethics Committee of Geneva University Hospitals (TLR patients) and the Ethics Committee of the University of Queensland (controls). The remaining subset of participants that completed the facial emotion task and full participant set that completed the bodily emotion task were approved by the Metro North Health Human Research Ethics Committee (TLR patients) and the Ethics Committee of the University of Queensland (controls). Participants gave their written informed consent to participate before beginning the experiment.

#### Facial emotion task

There were two variations of the facial emotion task. Of the TLR groups, 11 participants (6 LTLR, 5 RTLR) completed the one-back task outlined in Framorando et al. (2021), and this was matched in the control group (5). Stimuli were 30 grayscale photographs (236 × 236 pix) of 10 identities (five female) displaying fearful (10), happy (10), or neutral expressions (10), from the K-DEF database (Lundqvist et al., 1998). Faces were cropped to remove external features (e.g., ears/hair) and equated for luminance across all categories using ImageJ (Rasband, 2018). Additionally, 20 stimuli of common vegetables, adjusted to grayscale, and altered for size and luminance to match the face set were created.

This subset of the lobectomy group completed a one-back task. Participants were instructed to fixate on a central white fixation cross, which would be present for a random duration between 500 and 1,000 ms at the start of each trial. An image would then be presented centrally for 300 ms. A blank screen would then appear for 1,200 ms before the next trial began. Images were presented in a randomized order, and participants were instructed to make a key press when a stimulus was presented twice in immediate succession (these repetitions occurred on 10% of trials). Each of the 30 face images were repeated eight times, for a total presentation of 240 face trials (80 per emotion), and the 20 vegetable distractor images were repeated four times for a total of 80 presentations. The full experimental run was ∼25 min.

The remaining seven lobectomy participants (3 LTLR, 4 RTLR) and four control participants completed a facial emotion recognition task. Stimuli were 80 grayscale photographs (236 × 236 pix) of 40 identities (20 female) displaying fearful (20), or neutral (20) expressions, from the K-DEF database (Lundqvist et al., 1998). Faces were converted to grayscale, cropped to remove external features (e.g., ears/hair), and equated for luminance across all categories using ImageJ (Rasband, 2018).

This subset of the lobectomy group completed an emotion recognition task. Participants were instructed to fixate centrally. At the start of each trial a white fixation cross would appear for 600–1,000 ms. This was followed by a centrally presented face which would appear for 500 ms, followed by an 800 ms screen displaying a fixation cross. Participants were asked to indicate on a blank screen whether the face displayed a fearful or neutral expression with a key press (each indicated by a separate key). The participants’ response would initiate the next trial. Photographs were presented in random order, and each photograph was repeated ∼2.5 times for a total of 200 presentations (100 per emotion condition). The full experimental run was ∼6 min.

#### Bodily emotion task

Stimuli were 80 grayscale photographs (142 × 310 pix) of 40 identities (20 female) displaying fearful (20) or neutral (20) bodily expressions, from a standardized published database (BEAST; De Gelder and Van den Stock, 2011). Bodies were converted to grayscale, and faces were blurred to disguise facial expressions. Images were equated for luminance across all categories using ImageJ (Rasband, 2018).

Bodies were presented in a bodily emotion recognition task. Participants were instructed to fixate centrally. At the start of each trial, a white fixation cross would appear for 600–1,000 ms. This was followed by a centrally presented body which would appear for 500 ms, followed by an 800 ms screen displaying a fixation cross. Participants were asked to indicate on a response screen whether the body displayed a fearful or neutral expression with a key press (each indicated by a separate key). The participants’ response would initiate the next trial. Photographs were presented in random order, and each photograph was repeated ∼2.5 of times for a total of 200 presentations (100 per emotion condition). The full experimental run was ∼6 min.

#### Apparatus

The facial one-back task was conducted on a 21 inch monitor (Hewlett-Packard, LCD screen; refresh rate of 60 Hz), situated 115 cm from the subject. For the facial and bodily emotion, recognition task stimuli were presented on a 24 inch (ASUS LCD monitor model VG248QE, resolution: 1,920 × 1,080 pixels; refresh rate, 60 Hz), situated 115 cm from the subject.

Continuous EEG was acquired at 1,024 Hz using an AD-Box ActiveTwo amplifier and 64 equally spaced scalp electrodes referenced to CMS/DRL. Two external electrodes EOG were placed on the face in order to monitor eyeblinks and saccades (one on the outer canthus of the right eye and one above the right eyebrow). Triggers were time locked to stimulus onset, and timing was verified with the use of a photodiode during experiment preparation.

## Results

### EEG preprocessing

Data for the subset of participants that completed the one-back facial emotion task was preprocessed in Framorando et al. (2021). Additional data collected was preprocessed in accordance with the pipeline outlined in Framorando et al. (2021) to prevent any artifacts arising between the groups due to differences in data preparation. EEG data was preprocessed using the EEGLab (Delorme and Makeig, 2004) and ERPLab (Lopez-Calderon and Luck, 2014) toolboxes in MATLAB (2021). Electrodes with bad signals were interpolated using 3D spherical splines. Data was downsampled to 512 Hz, and rereferenced offline to Cz. Cz was selected as the reference as opposed to the more commonly used average of all electrodes to prevent the introduction of noise from scar tissue around the temporal site into the whole dataset, as has been done in previous temporal lobectomy EEG studies (Framorando et al., 2021). Data was filtered with a low cutoff of 30 Hz and a high cutoff of 0.1 Hz. Data was segmented into time-locked epochs 100 ms before and 350 ms after stimulus onset, with a 100 ms baseline correction. A channel was created using two facial electrodes to isolate trials that included eyeblinks and movements. Artifact rejection was conducted whereby any trial from this computed external channel or the 64-electrode channels that exceeded ±100 µV during the segmented epoch were excluded from analysis to account for eye movements, blinks, and muscle movements. Each trial was then combined to create averages for each condition per participant for use in MUA.

### Mass univariate analysis

Separate cluster-based permutation *t* test MUAs were conducted for each group (LTLR/RTLR/control) for each of the two stimuli comparisons (bodily emotion and facial emotion which combined two tasks—one-back and emotion recognition) to compare neural responses with neutral and fearful stimuli presentations. The epochs for comparison selected were based on peaks identified through the GFP (global field power) of the GAV (grand average). Isolated epochs were examined instead of the full-time range to maximize the statistical power, considering the small clinical sample size. MUA was conducted using the Mass Univariate ERP Toolbox (Groppe et al., 2011).

Average ERPs for each condition for each participant were examined through a repeated-measures, two-tailed cluster-based permutation test based on the cluster mass statistic (Bullmore et al., 1999) using a family-wise alpha level of *p *< 0.05. Time points between 90 and 350 ms at all 64-electrode sites were included in the test (divided into four temporal epochs). Repeated-measures *t* tests were performed for each comparison using the original data and 2,500 random within-subjects permutations of the data. For each permutation, all *t*-scores with an uncorrected *p* value of <0.05 were formed into clusters with any spatially or temporally neighboring such *t*-scores, where electrodes within 0.41 units (∼3.65 cm) proximity of each other were considered spatial neighbors and adjacent time points were considered temporal neighbors. Mass of each cluster was calculated as the sum of *t*-scores within that cluster, and the most extreme cluster mass in each of the four sets of tests was recorded and used to estimate the distribution of the null hypothesis. *p* values were derived from the percentile ranking of the permutation mass of each cluster.

### Facial emotion task

**Figure 1.**
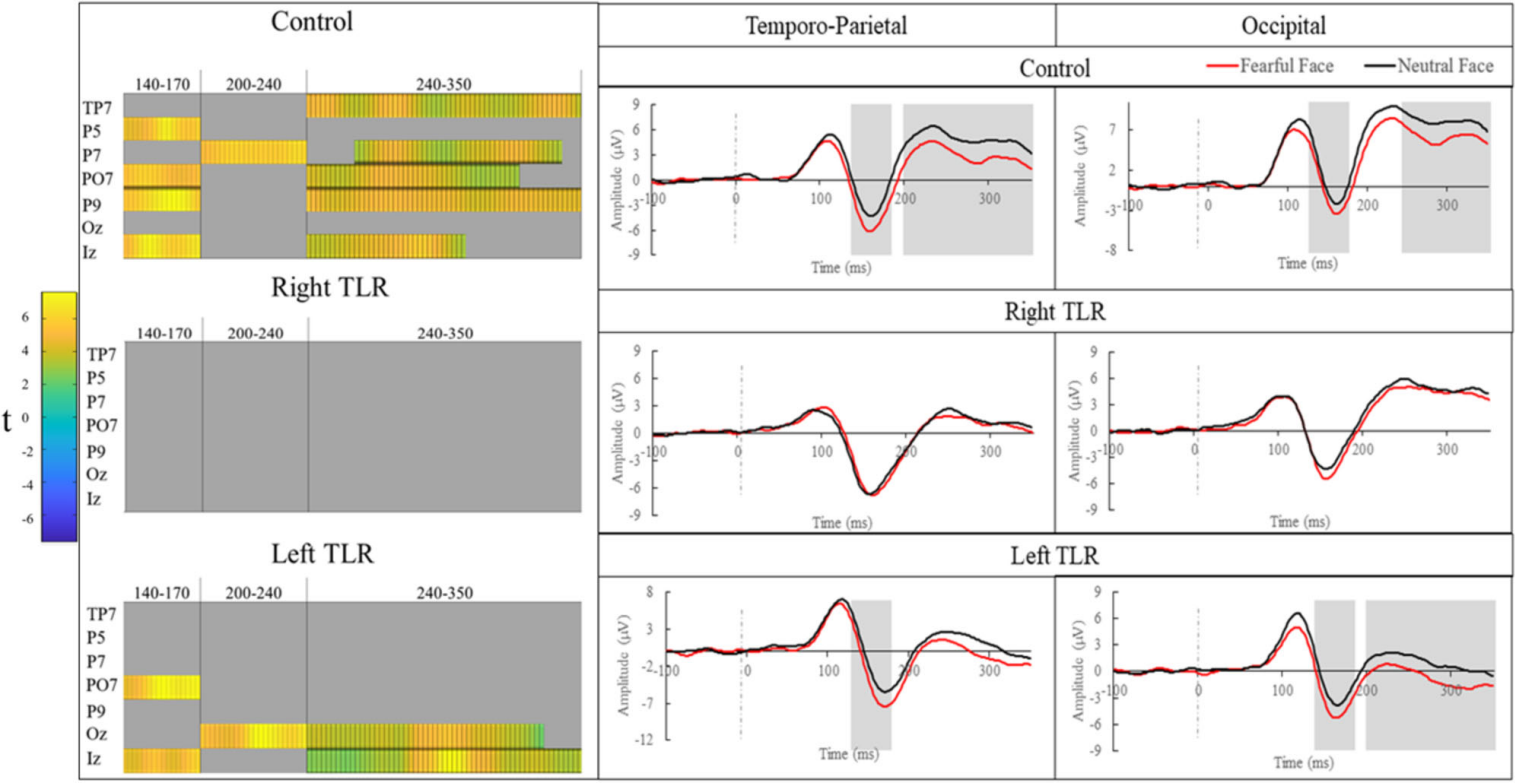
Results for the MUA facial emotion comparison. Left, Condensed raster plots for epochs and channels following the MUA cluster permutation analysis. Note all 64 electrodes were included in the analysis but only channels that were significantly different in any of the three groups have been shown in this representation. Orange areas indicate epochs and channels where a significant emotion based difference was found. Right, ERPs represent the pooling of clusters of electrodes that were found to be significantly different in any of the three groups for the facial expression comparison. These have been separated into temporoparietal (TP7, P5, P7, P9, PO7) and occipital (Oz, Iz) groupings. Shaded areas indicate where at least one of the pooled electrodes were found to be significantly different between the emotion condition for that group.

Epochs identified through appraisal of the grand average GFP were 90–120 ms, 140–170 ms, 200–240 ms, and 240–350 ms. All significant differences are represented in Figure 1.

#### Control

The first difference between facial emotion emerged for the control group at the 140–170 ms time window over electrodes P5, P9, PO7, and Iz (all *t*’s > 3.57, *p*’s < 0.05, test-wise *p *= 0.007). This was followed by a difference at the window 200–240 ms over electrode P7 (*t*’s > 3.44, *p*’s < 0.05, test-wise *p *= 0.009) and at the 240–350 ms time window over electrodes TP7, P7, P9, PO7, and Iz (all *t*’s > 2.31, *p*’s < 0.05, test-wise *p* = 0.049).

#### Right TLR

No significant clusters emerged at any time windows over any electrode sites.

#### Left TLR

The first difference emerged at the 140–170 ms time window over electrodes PO7 and Iz (all *t*’s > 3.29, all *p*’s < 0.05, test-wise *p *= 0.011). This was followed by a difference at 200–240 ms over Oz (all *t*’s > 2.69, all *p*’s < 0.05, test-wise *p* = 0.027) and at 240–350 ms over Iz and Oz (all *t*’s > 2.38, all *p*’s < 0.05, test-wise *p *= 0.044).

### Bodily emotion task

**Figure 2.**
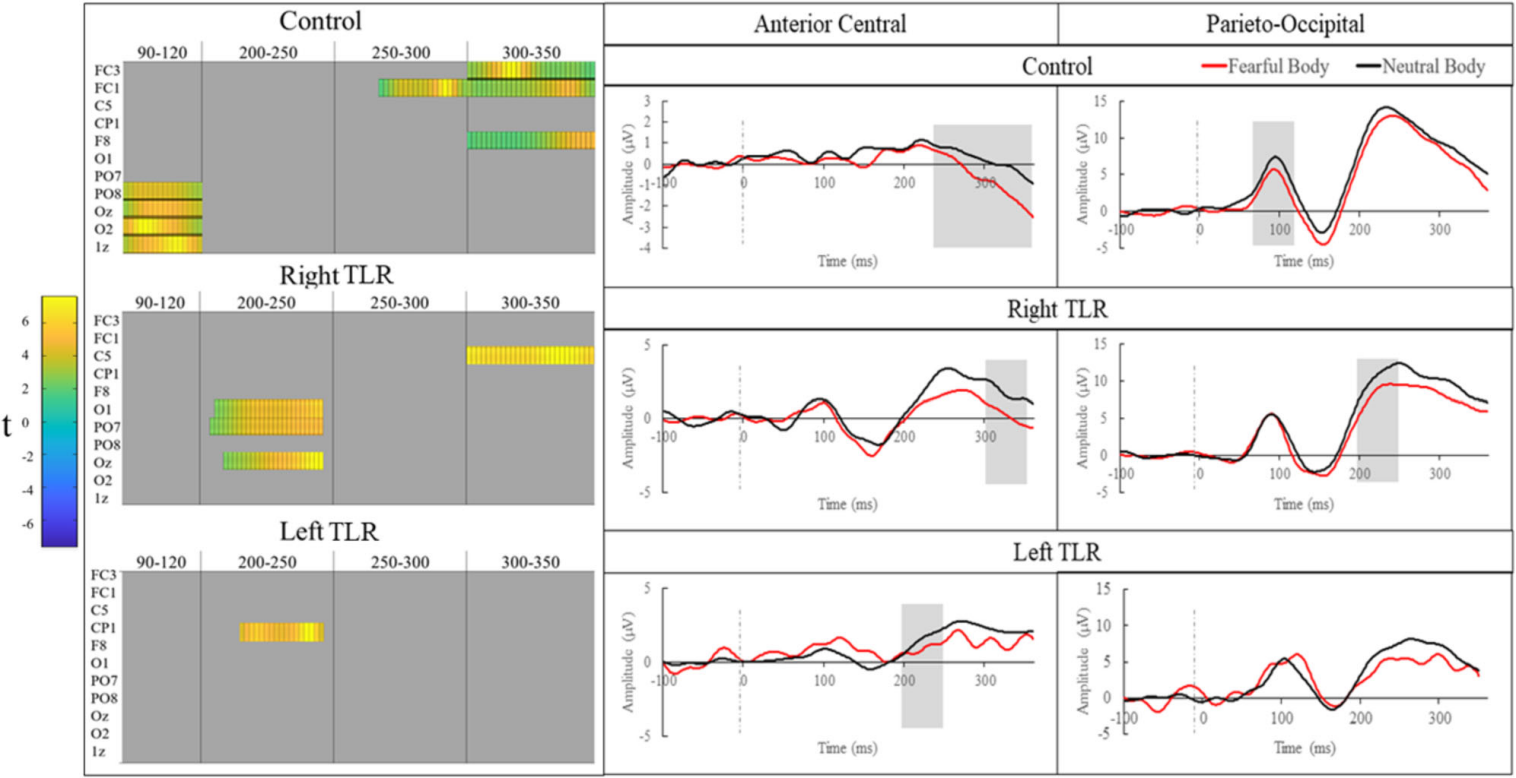
Results for the MUA bodily emotion comparison. Left, Condensed raster plots for epochs and channels following the MUA cluster permutation analysis. Note all 64 electrodes were included in the analysis but only channels that were significantly different in any of the three groups have been shown in this representation. Orange areas indicate epochs and channels where a significant emotion-based difference was found. Right, ERPs represent the pooling of clusters of electrodes that were found to be significantly different in any of the three groups for the bodily expression comparison. These have been separated into anterior-central (CP1, C5, FC1, FC3, F8) and parieto-occipital (PO8, PO7, O1, O2, Oz, Iz) groupings. Shaded areas indicate where at least one of the pooled electrodes were found to be significantly different between the emotion condition for that group.

Epochs identified through appraisal of the grand average GFP were 90–120, 130–150, 200–250, and 250–350 ms. All significant differences are represented in Figure 2.

#### Control

The first difference between bodily emotion emerged for the control group at the 90–120 ms time window, over electrodes Oz, Iz, PO8, and O2 (*t*’s > , *p*’s < 0.05). The next difference was evident at the 250–300 ms time window over electrode FC1 (*t*’s > , *p*’s < 0.05) and then from the 300–350 ms time window over electrodes FC3, FC1, and F8 (*t*’s > , *p*’s < 0.05).

#### Right TLR

The first difference of bodily emotion emerged at the 200–250 ms time window over electrodes PO7, O1, and Oz (all *t*’s > 2.45, *p*’s < 0.05). A later difference emerged at the 300–350 ms time window, over electrode C5 (*t*’s > 3.29, *p*’s < 0.05).

#### Left TLR

The only difference between bodily emotion emerged at the 200–250 ms window over electrode CP1 (all *t*’s > 2.76, *p*’s < 0.05).

### ERP amplitude analysis

#### Facial emotion task

At 140–170 ms (electrodes P5, PO7, P9, Iz), there was a main effect of emotion, *F*_(1,8)_ = 11.91, *p* = 0.009, *ƞ_p_*^2^ = 0.598, such that fearful faces (*M* = −6.29, SD = 4.84) evoked a greater amplitude than neutral (*M* = −5.45, SD = 4.17). No other effects were significant.

At 200–240 ms (electrodes P7, Oz), there was a main effect of emotion, *F*_(1,7)_ = 5.95, *p* = 0.041, *ƞ_p_*^2 ^= 0.427, such that fearful faces (*M* = 2.75, SD = 3.96) evoked smaller amplitudes than neutral (*M* = 3.51, SD = 4.58). There was a main effect of group, *F*_(2,16)_ = 6.36, *p* = 0.009, *ƞ_p_*^2 ^= 0.443, follow-up tests revealed a significant difference between the control (*M* = 5.92, SD = 2.89) and LTLR (*M* = 0.65, SD = 2.86) groups, *t* = 3.78, *p*_bonf _= 0.016, Cohen’s *d* = 1.34, mean difference = 5.26, but no other differences between clinical groups.

At 240–350 ms (electrodes TP7, P7, PO7, P9, Oz, Iz), there was a main effect of emotion, *F*_(1,7)_ = 18.70, *p* = 0.003, *ƞ_p_*^2 ^= 0.700, such that fearful faces (*M* = 0.79, SD = 4.09) evoked smaller amplitudes than neutral (*M* = 2.07, SD = 4.75). There was a main effect of group, *F*_(2,16)_ = 6.84, *p* = 0.007, *ƞ_p_*^2 ^= 0.461, and follow-up tests again revealed a difference between control (*M* = 3.68, SD = 4.19) and LTLR (*M* = −1.65, SD = 2.53) groups, *t* = 3.32, *p*_bonf _= 0.032, Cohen’s *d* = 1.32, mean difference = 5.33, but no other differences between clinical groups.

#### Bodily emotion task

To assess the differences between groups beyond the MUA conducted, amplitude analysis was conducted on the epochs identified in the initial analysis. For the below follow-up analyses, 3 (group: control, LTLR, RTLR) × 2 (emotion: fearful, neutral) repeated-measures ANOVAs were conducted. Bonferroni’s corrections were applied to any follow-up tests.

At 90–120 ms (electrodes PO8, Oz, O2, Iz), there was a main effect of emotion, *F*_(1,7)_ = 6.53, *p* = 0.038, *ƞ_p_*^2 ^= 0.482, such that fearful bodies (*M* = 3.39, SD = 3.80) evoked a smaller amplitude than neutral (*M* = 4.31, SD = 3.27). There was no effect of clinical group and no interaction.

At 200–250 ms (electrodes O1, PO7, Oz, CP1), there was a main effect of group *F*_(2,14)_ = 3.95, *p* = 0.043, *ƞ_p_*^2 ^= 0.361; however, follow-up tests did not reveal differences (all *p*’s > 0.050). There was a main effect of emotion, *F*_(1,7)_ = 24.64, *p* = 0.002, *ƞ_p_*^2 ^= 0.779, and fearful bodies (*M* = 7.26, SD = 4.41) evoked smaller amplitudes than neutral (*M* = 8.78, SD = 4.52). There was no interaction.

At 250–300 ms (electrodes FC1), there were no significant effects or interactions.

At 300–350 ms (electrodes FC3, FC1, F8, C5), there was a main effect of emotion, *F*_(1,7)_ = 6.36, *p* = 0.040, *ƞ_p_*^2 ^= 0.476, whereby fearful bodies (*M* = −1.63, SD = 3.37) evoked smaller amplitudes than neutral (*M* = −0.81, SD = 3.27). No other effects were significant.

## Discussion

In this paper we investigated how two groups of patients with TLR comprising the amygdala and in the majority of instances the hippocampus and temporal pole encoded facial and bodily expressions using time-resolved EEG data. This approach yielded a number of key insights. For readability, we have included labels that pertain to significant temporal and spatial epoch clusters. First, the comparison of emotional facial expressions revealed significant differences in the neural processing of fearful and neutral faces for healthy controls from 140 to 170 ms over temporoparietal and occipital electrodes (N170) and then at 200–350 ms over temporoparietal and 240–350 ms over occipital electrodes (posterior P2). A similar but more restricted pattern of activity was observed for the clinical group that had undergone left TLR, with temporoparietal and occipital differences from 140 to 170 ms (N170) and occipital differences to emotional faces from 200 to 350 ms (posterior P2). In contrast, no significant neural differences were found for fearful compared with neutral faces in these time ranges for the right TLR group consistent with the findings from Framorando et al. (2021).

Second, the comparison of emotional bodily expressions revealed that, for healthy controls the first difference in the neural processing of fearful and neutral bodies was observed at 90–120 ms over parieto-occipital electrodes (P1), followed by anterior-central differences to fearful and neutral faces from 250 to 350 ms (anterior P2). Notably, the earliest emotion-based bodily difference seen in the control group, was absent in both TLR groups. Rather, the right TLR group showed a different emotion-based neural response to bodies at 200–250 ms over parieto-occipital electrodes and 300–350 ms over anterior central electrodes (posterior P2 and anterior P2). Meanwhile, the only difference observed for the left TLR group was at 200–250 ms over anterior central electrodes (anterior P2). This pattern of results indicates that, while the right amygdala is crucial for the initial processing of emotional faces, unilateral TLR impairs the earliest stages of emotional body perception, regardless of the hemisphere that is targeted in surgery.

While the MUA revealed that emotion-driven patterns appeared to differ between TLR and healthy groups, the typical ERP amplitude analysis did not indicate any between group interactions. The typical main effects of emotion were present for both face and body tasks (Stekelenburg and de Gelder, 2004; van Heijnsbergen et al., 2007; Hinojosa et al., 2015). However, the only between group differences evident were at mid-late processing stages for the facial task, between healthy and LTLR groups. While both groups showed emotion-related differences in amplitude, the overall amplitude to faces of both emotions for the LTLR group was dampened at 200–240 and 240–350 ms. We note, however, that these results are based on a small clinical group, and, thus there may not be sufficient power to detect meaningful interactions.

Considering the processing of emotional faces, for healthy controls the first emotion-based difference matched the time window and parieto-occipital region corresponding to the N170 (Hinojosa et al., 2015). The activation of this component is expected because the N170 is well established as the first face-specific component that is modulated by facial expression (Hinojosa et al., 2015). In the current data, this was followed by sustained emotion-based differential activation over temporoparietal and occipital cortices from ∼200 ms onward on the second positive peak. This temporo-posterior P2 has been linked to higher-stage cognitive processes (Campanella et al., 2002), such as stimulus evaluation and the mental representation of the emotional content which can contribute in a task-dependent fashion to adaptive behaviors. Therefore, the results from the control group are broadly consistent with the current scientific record and provide a healthy baseline to compare emotional face processing post-TLR.

Here, we show the complete absence of emotion-based activation in the right TLR group (from 90 to 350 ms). The absence of the earliest N170 ms deflection for the right TLR group is followed by the lack of any mid-stage differences. This indicates that the resected regions play a critical role in contributing to the initial encoding of relevant emotional information in faces. The amygdala responds rapidly to emotional faces (within ∼70 ms; Inagaki et al., 2023; Méndez-Bértolo et al., 2016; Weidner et al., 2022) and continually contributes via feedback and feedforward connections. The absence of an N170 modulation by emotion is likely a reflection of an absence of amygdala input to face-selective right hemispheric cortical regions. Other TLR research shows such impaired early emotion modulations following right resection (Rotshtein et al., 2010; Mielke et al., 2022; Kissler et al., 2023). The absence of a subsequent differential response to facial expressions builds on previous research showing that subcortical bodies modulate extrastriate cortical responses to fearful faces (Morris et al., 1996; Vuilleumier et al., 2004; Liu et al., 2022). Further, previous studies of patients with right TLR have indicated that the loss of the right amygdala reduces the response of the right fusiform lingual gyrus to faces (Reisch et al., 2022a) and the response of the visual cortex to affective scenes (Reisch et al., 2022b). By extension, the present findings indicate that the resected areas appear crucial for early affective face processing and uniquely contributes to mid-stage processing via cortical connections (Kapp et al., 1994; Armony et al., 1998; Cahill and McGaugh, 1998; Anderson and Phelps, 2001; Amaral et al., 2003; Hung et al., 2010; Vrticka et al., 2013; Kohno et al., 2015). Indeed, amygdala sclerosis is related to absent cortical activity to faces as typically seen in healthy populations (Vuilleumier et al., 2004). It is likely that later emotion-driven differences would emerge for the right TLR group, as retained emotion recognition is often reported (Reisch et al., 2022a; Kissler et al., 2023), and emotion differences comparable with controls are seen in ERPs from 400 ms onward when viewing affective pictures (Mielke et al., 2022).

The maintenance of early and mid-stage emotional-based differences in the left TLR, but absent in the right TLR population, highlights two interesting points: firstly, that there is a functional lateralization for emotional face processing, and secondly, that the initial stage of structural face encoding is seemingly necessary for the occurrence of mid-stage processes. Regarding the first point, this aligns with a large body of literature showing lateralized-right dominance in face processing (Kanwisher et al., 1997; Bukowski et al., 2013; Corrow et al., 2016). The present findings indicate that this hemispheric dominance extends to facial emotion processing (Gainotti, 2012) or that the structural encoding facilitated by the right amygdala is crucial for subsequent cortical emotion processing (Burton et al., 2003; Bertini et al., 2019). On the second point, the presence of later emotion differences in the left TLR group (posterior P2 from 200 ms onward) following the spared effect at N170 could indicate that facial emotion processing is hierarchical with later stages gated by earlier ones (Bruce and Young, 1986; Haxby et al., 2000).

It has been suggested that emotion and in particular threat are encoded automatically—a process posited to be reliant on the amygdala (Compton, 2003; Öhman, 2005; Phelps and LeDoux, 2005). However, task demands such as the relevance of the face or emotion could impact the extent to which the amygdala is recruited in visual perception. Intracranial EEG evidence has previously suggested that the amygdala is responsive to the emotional content of faces (from 130 ms) regardless of task relevance (Hadj-Bouziane et al., 2012). This has been corroborated by EEG studies showing task independent responses to emotional faces at early stages (Schupp et al., 2003, 2006; Pourtois et al., 2010; Schindler and Kissler, 2016; Schindler et al., 2020). In contrast, other electrophysiological recordings have suggested that mid-latency scalp (Weidner et al., 2022) and amygdala (Krolak-Salmon et al., 2004; Pourtois et al., 2010) responses to emotion are modulated by task relevance. A systematic review of the topic reported that task relevance did not alter emotion modulations at early ERP components such as EPN and N170 but did at later components like P3 and LPP (Schindler and Bublatzky, 2020; also see Krolak-Salmon et al., 2001). The implication is that bottom-up and top-down processes may interact with each other and that the amygdala could have bidirectional connections with attention-related cortical regions (Vuilleumier, 2005), as well as the ventral visual system (Morris et al., 1998; Pessoa et al., 2002; Krolak-Salmon et al., 2004). These bidirectional connections are particularly relevant to the present study because the face task was split into two subsamples, one an emotion-salient task (emotion recognition) and the other a perception/memory task (one-back). Both tasks necessitated sustained attention and perceptual encoding but varied in task demands and thus may have recruited the amygdala differently via different connections. Research has indicated that in a healthy sample, amygdala activity would be evoked to a greater degree during emotion recognition compared with a one-back task, due to the salience of the emotional content. However, this does not undermine our findings, as task type was matched across all groups (i.e., the same proportion of the LTLR, RTLR, and healthy samples completed both tasks). The combination of the two tasks in the present study therefore can be used to examine differences in affective processing postresection. Further research will be required to examine the impact of resection on the processing of emotional stimuli when task relevance varies.

Considering the processing of emotional bodies, for controls we observed the earliest emotion-based differences emerging over occipital electrodes at 90 ms (P1), followed by anterior-central activation from ∼250 to 350 ms (anterior P2). The occipital activation from 90 to 120 ms matches P100 visual activation (Batty and Taylor, 2003). P100 reflects visual encoding as well as aspects of attention. In healthy groups, P100 emotion modulations have been found elsewhere for bodies (van Heijnsbergen et al., 2007), followed by mid-late stage emotion modulated fronto-central negativity corresponding to our later emotion-related negative deflections on P2 (Stekelenburg and de Gelder, 2004). Importantly the earliest emotion-differential response was absent in both TLR groups, suggesting that the P100 is reflective of more than just the processing of low-level contributions. The absence of this early-stage difference in both groups suggests that the earliest neural response to bodies requires bilateral medial temporal activation, while contralateral activation or cortical activation is sufficient for later responses to bodies. Importantly, bodies have been shown to be represented in the primate temporal cortex—a site resected in the majority of the clinical group (Vogels, 2022). It is thus likely that temporal body-specific contributions are impaired in those cases. For both TLR groups later stage differences at P2 were present, which could reflect intact mid-late stage cognitive processes such as appraisal or judgment. This indicates that later stage processes that inform cognition do not rely on the initial neural response to bodily expressions.

Although studies of emotion frequently use static stimuli, it has been claimed that images of human figures may imply motion and as such might activate areas sensitive to biological motion in the brain. Without motion, the body processing network is thought to involve the STS and the extrastriate body area (de Gelder, 2006), with the processing of bodily expressions additionally activating the amygdala and fusiform gyrus (Hadjikhani and de Gelder, 2003). Emotional body expressions, in comparison with neutral representations, can indicate implied motion such as approach or avoidance. Implied motion activates medial temporal and medial superior temporal cortices (Kourtzi and Kanwisher, 2000). ERP research investigating lateralization of emotional body processing has found stronger right lateralized effects for emotional content, and stronger left effects for implied movement (Borhani et al., 2015), potentially explaining the required bilateral amygdala contribution for the early P1 difference.

The present results speak to the multiplexed contribution of the medial temporal areas to emotion processing. While it is evident that face and body emotion processing activate different networks (de Gelder et al., 2004; van de Riet et al., 2009), these networks have shared components, such as the amygdala (van de Riet et al., 2009). However, it has proven difficult to characterize the unique contributions of the amygdala to face and body emotion processing. We argue that by comparing the pattern of results from different groups of TLR patients, we can distil similarities and differences in the face and body processing networks. For example, our results demonstrate that medial temporal activation is necessary for early emotion-based electrophysiological differences for both categories.

While all clinical participants underwent amygdala resection, often adjacent areas were also affected. For the facial task, the additional resected sites included portions of the temporal lobe in 14 out of 18 clinical participants and the hippocampus in 14 out of 18 clinical participants. For the body task, the additional resected sites included portions of the temporal lobe in 14 out of 16 clinical participants, and the hippocampus in 8 out of 16 clinical participants. Temporal lobe epilepsy has been linked to deficits in fear-specific emotion recognition (Nineuil et al., 2023). However, these deficits are more pronounced than in cases of lateral temporal lobe epilepsy, suggesting that medial temporal structures like the amygdala play a unique role in fear recognition (Nineuil et al., 2023). Interestingly, hippocampal volume is positively associated with the speed of facial emotion identification (Szymkowicz et al., 2016). Therefore, it remains possible that these additional resections may have directly or indirectly impacted some of our results. One point of contention related to their pathologies is that individuals with epilepsy may be subject to reorganization of the neural networks in the medial temporal region involved in emotion processing. Indeed, recurrent seizures can lead to sclerosis or impaired development at the focal site and surrounding areas. Epilepsy-related sclerosis has been related to the degree of electrophysiological impairment in emotional face processing (Rotshtein et al., 2010). The age of seizure onset is related to the degree of damage, and impaired behavioral emotion recognition has been found for those with earlier but not later onset ages (McClelland et al., 2006). While amygdala activation is still evident for fearful faces in groups with medial temporal lobe epilepsy (Schacher et al., 2006), the lateralization of this activation can shift to the hemisphere contralateral to the focal seizure origin (Riley et al., 2015) and must be acknowledged when considering this clinical group. In this way, it is also possible that some differences observed between the face and body groups—which used different participant subsets—may have been due to variations in seizure-related damage or functional reorganization across individuals. For this reason, it is imperative that future research includes measures of the age of seizure onset as this could account for both functional variation between individuals and discrepancies in the lobectomy literature. Additionally, the assessment of both behavioral and neural measures pre- and postoperatively would provide an understanding of baseline processing and the functional outcomes related to surgical intervention. The present results shed new light on the complex role of medial temporal structures in emotion processing. Our results reveal the unique roles of these structures at the earliest emotion-sensitive epochs for face and body processing. Additionally, the findings demonstrate that activity in the unilateral right resected area is necessary for distinguishing different facial expressions, while contralateral activation is necessary for the earliest emotional body processing. Although these results alone cannot distinguish between the importance of different medial structures and temporal cortices, there have been previous indications that the amygdala is particularly important to these processes (Cecere et al., 2014; Reisch et al., 2022a). The difference in neural activation in response to faces and bodies following TLR indicates that while faces and bodies are processed by similar brain networks, there are key points of divergence that can function independently.

## Data Availability

Code and data are available at https://osf.io/jwm6t/.

## Synthesis

Reviewing Editor: Alexander Soutschek, Ludwig-Maximilians-Universitat Munchen Decisions are customarily a result of the Reviewing Editor and the peer reviewers coming together and discussing their recommendations until a consensus is reached. When revisions are invited, a fact-based synthesis statement explaining their decision and outlining what is needed to prepare a revision will be listed below. The following reviewer(s) agreed to reveal their identity: Marta Garrido.

Reviewer #1

General comment:

This is a very nice paper demonstrating a causal role for the right temporal lobe in emotional face processing. Furthermore it shows that for emotional body perception the bilateral temporal lobe is key.

Major comments:

1) The study makes some critical claims about the functional role of the right amygdala in face processing and bilateral amygdala in emotional body perception. This is based on data from patients with TLR, which includes the amygdala. Do the TLRs only include the amygdala or are other temporal lobe areas involved in the resection? If there are other areas, how confident can we be about this function being specific to the amygdala?

2) Given that the groups that underwent the task for bodies and faces were slightly different, how much of the differences we see in the data could be driven by small differences in the patient sample? I'm curious as to why weren't both tasks run in the same patient?

3) Please discuss potential implication of using different tasks for different subsets of patients in the face emotion task. Could task set modulate the ERPs data shown?

4) I would love to see what the behavioural data look like for emotion recognition across face and body tasks. Is the task performance related to the EEG data? If so, this would make the argument around emotion discrimination being related to right/bilateral amygdala (or temporal lobe - see comment 1 above) a lot stronger.

Minor:

5)Page 14, "that the earliest stage of structural face encoding necessitates mid-stage processes" I'm a bit confused by this statement. Do the authors mean the reverse, that the mid-stage necessitates the earliest stage?

6) Define GFP and GAV

Reviewer #2

In this manuscript, the authors investigate the potential role of the amygdala in visual emotional processing. They do this by comparing patients with left or right unilateral temporal lobe resections to a control group regarding their electrophysiological indicators of emotion processing in both faces and bodies. Given that systematic resection studies on this topic are still sparse, this paper adds a very valuable contribution to the field. Furthermore, the use of emotional bodies as stimulus materials in such a study is a novel and interesting approach towards a more holistic overview of emotion processing. However, there are a few concerns that I would like to raise that could improve the overall paper:

First, I would advise the authors to de-emphasize the idea about subcortical emotion signals, as the data does not really allow any inferences about this. Like the introduction, the discussion emphasized the idea of subcortical fear signals but bases this off the fact that right TLR patients were lacking differential effects around 140-170 ms. This is not a time-window that is commonly associated with subcortical signals and as much as this is a very interesting topic of research, I do not think that the data allow for strong assumptions about these pathways. They do, however, imply cortico-amygdalar interactions that could be emphasized more throughout the manuscript.

I wonder why the authors chose to use two separate tasks for investigating face processing. In my opinion, this limits the comparability of the face and body experiments since the tasks were partly different. Recognition of emotional categories versus simple n-back tasks will likely trigger different attentional mechanisms that also potentially recruits the amygdala differently. I would be interested in seeing whether results differ between the two face tasks, although the small sample might hinder this comparison. This should at least be acknowledged in the limitations.

Regarding the analyses, I was expecting a direct group comparison in combination with the emotion comparison but from what I understood, the emotion categories were compared within each group separately? Given that it would be interesting to see resection-specific changes compared to a control group, I would be interested in seeing how emotion processing differs between the groups.

I think the cluster-based analysis approach on pre-defined time-windows is not necessarily the most useful approach in this case, as the authors still basically investigated pre-defined ERPs. I would advise to ditch the cluster-based approach while keeping the non-parametric permutations, investigating ERPs of interest directly in their respective channel groups. This would also facilitate the comparability to previous resection studies and the readability of the effects in the discussion (rather than stating "the effect from 90-120 ms over parieto-occipital electrodes", one could also refer to it as the P1). I am aware that this categorization is done later in the discussion, but in my opinion, this should be done from the start.

Some minor points:

Was the offline reference really the Cz electrode, or the common average?

As such samples are relatively rare, I would be interested to see some more explorations of the data. E.g., I would be curious to know whether the found effects are modulated by any clinical variables, i.e., at what age did the patients undergo the resection, how much time was passed since the resection etc., particularly because the authors acknowledge potential re-organizational mechanisms in their limitations.

I like the figures; they are very informative and easy to grasp.

Overall, I think that this is a very interesting study, but the authors should be careful with their interpretation. More advanced analyses would be justified to investigate this dataset in its full potential.
